# Case Report: Systemic Inflammatory Response and Fast Recovery in a Pediatric Patient With COVID-19

**DOI:** 10.3389/fimmu.2020.01665

**Published:** 2020-07-03

**Authors:** Adam Klocperk, Zuzana Parackova, Jitka Dissou, Hana Malcova, Petr Pavlicek, Tomas Vymazal, Pavla Dolezalova, Anna Sediva

**Affiliations:** ^1^Department of Immunology, 2nd Faculty of Medicine, Charles University in Prague and University Hospital in Motol, Prague, Czechia; ^2^Emergency Department for Children, University Hospital in Motol, Prague, Czechia; ^3^Department of Children and Adult Rheumatology, University Hospital in Motol, Prague, Czechia; ^4^Department of Anesthesiology and Intensive Care Medicine, 2nd Faculty of Medicine, Charles University in Prague and University Hospital in Motol, Prague, Czechia; ^5^Department of Paediatrics and Adolescent Medicine, Centre for Paediatric Rheumatology and Autoinflammatory Diseases, 1st Faculty of Medicine, General University Hospital in Prague, Charles University in Prague, Prague, Czechia

**Keywords:** COVID-19, SARS-CoV-2, PIMS-TS, innate immunity, hemophagocytic lymphohistiocytosis, macrophage activation syndrome, pediatric, neutrophil

## Abstract

We report a case of an 8-year-old girl who underwent a SARS-CoV-2 infection manifesting with atypical symptoms spearheaded by abdominal discomfort and systemic inflammation and partially mimicking hemophagocytic lymphohistiocytosis (HLH) or macrophage activation syndrome (MAS), which however did not fulfill the HLH/MAS diagnostic criteria. In this case of what has since been described as Pediatric Inflammatory Multisystem Syndrome Temporally associated with SARS-COV-2 (PIMS-TS) we documented excellent clinical response to immunosuppression with systemic corticosteroids and intravenous immunoglobulins. We show a detailed longitudinal development of neutrophil immunophenotype which suggests activation and engagement of neutrophils during PIMS-TS with compensatory contraction of the response and contra-regulation of neutrophil phenotype during recovery.

## Introduction

The recently emerged SARS-CoV-2 virus causes pneumonia and, in severe cases, acute respiratory distress syndrome in adults, but its clinical picture can be markedly different in children, most of whom undergo only a mild course of the disease (1–3). However, several recently published papers summarized a novel presentation of pediatric COVID-19, where the infection triggered a hyperinflammatory state provisionally labeled Pediatric Inflammatory Multisystem Syndrome Temporally associated with SARS-COV-2 (PIMS*-*TS), rather than the more commonly self-limited respiratory symptoms (4–6). Initial cohort descriptions are now starting to appear (7) which document abdominal discomfort, rash, and systemic inflammation as main symptoms of PIMS-TS and suggest good recovery with corticosteroid and intravenous immunoglobulin treatment. Detailed description of individual cases remains sparse, however, and our knowledge of the underlying immunopathology is still limited.

## Case Description, Diagnostic Assessment, Therapeutic Intervention, Follow-Up and Outcomes

**Figure 1:**
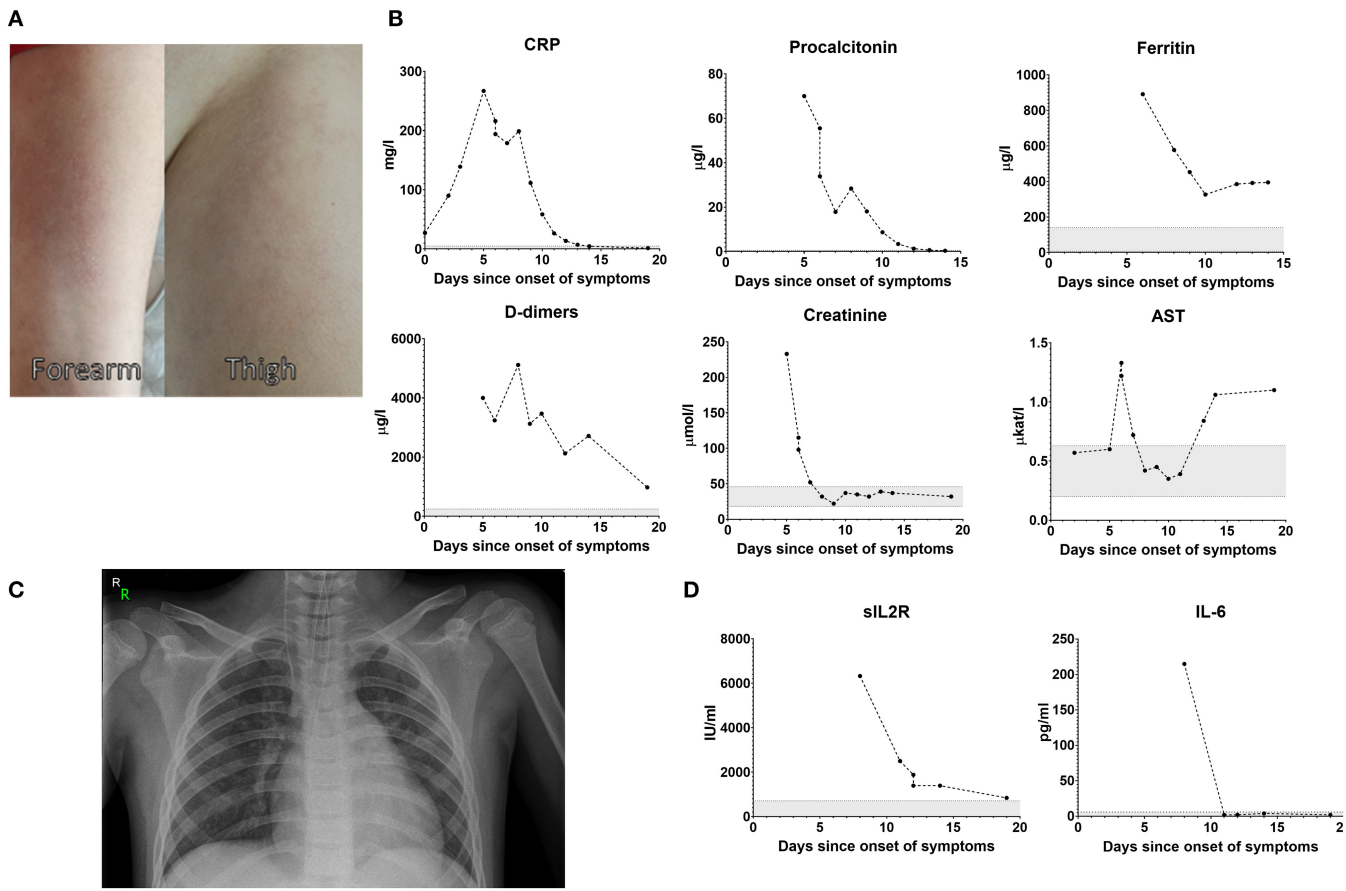
Exanthema on day 12 **(A)**. Blood biochemistry and markers of inflammation over the course of the disease **(B)**. Chest X-ray on day 6 showing only mild signs of hypoventilation in the retrocardiac region with no infiltration or consolidation **(C)**. Soluble IL-2 receptor and plasma IL-6 levels **(D)**.

**Figure 2:**
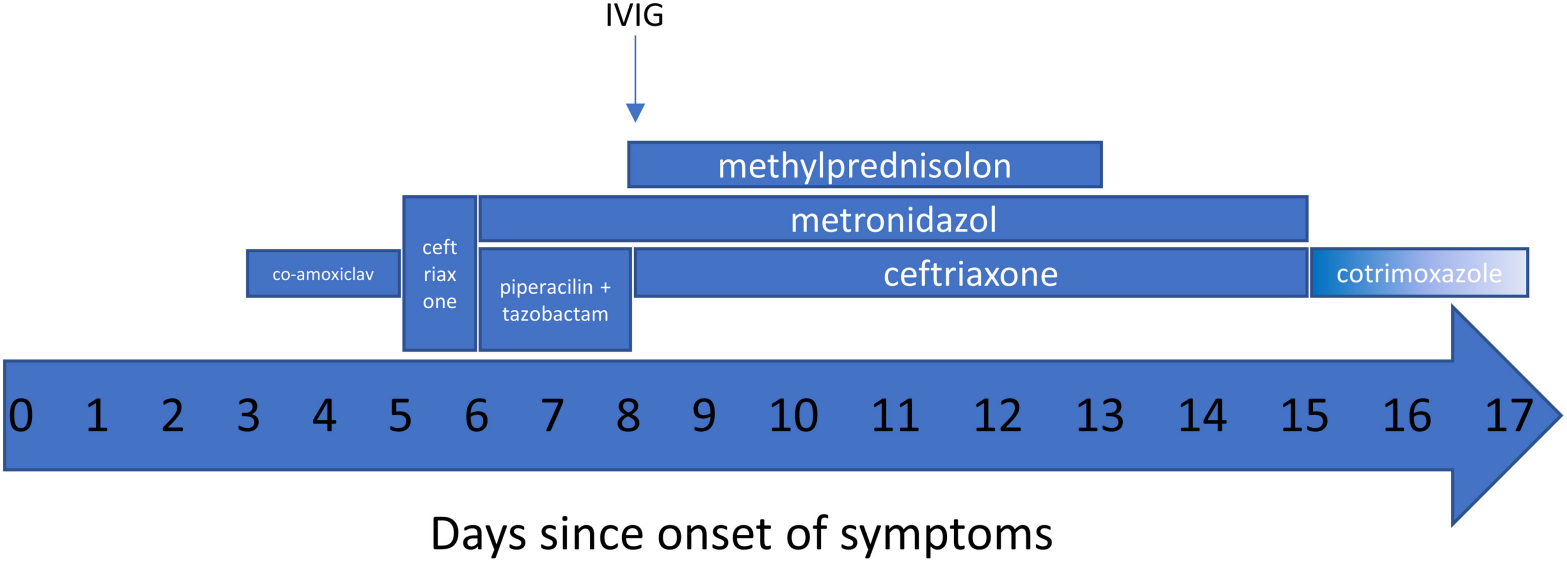
Timeline of main pharmacologic interventions.

Here we report a case of an 8-year-old girl who manifested with fever (>40°C), headache, abdominal pain, vomiting, diarrhea, and diffuse itchy maculo-papular rash (Figure 1A), but no signs of respiratory involvement. Her condition deteriorated quickly despite antibiotic therapy (Figure 2), necessitating hospital admission 5 days after onset of the disease. At admission she had high inflammatory markers (Figure 1B), elevated D-dimers, urea, creatinine, liver enzymes, troponin, and proNT-BNP. No microbiological (blood culture, panbacterial 16S PCR, herpes family PCR, endotracheal aspirate culture, viral and atypical pneumonia PCR, urine culture) or imaging tests (for chest X-ray, see Figure 1C) could explain all her symptoms. Abdominal ultrasound suggestive of paralytic ileus with appendicitis and overall worsening of clinical status prompted an empirical exchange of antibiotics and abdominal surgery on day 6, revealing only mild serous peritonitis.

After the discontinuation of sedation, her consciousness deteriorated toward Glasgow coma scale of 7–8, she developed dry cough and tested positive for nasopharyngeal SARS-CoV-2 PCR and virus-specific IgG.

The persistent elevation of CRP (199 mg/l), procalcitonin (28.4 μg/l), soluble IL-2 receptor (6,326 IU/ml, Figure 1D), ferritin (577 μg/l), and history of juvenile idiopathic arthritis (oligoarticular subtype, currently inactive without therapy) lead to suspicion of viral-induced macrophage activation syndrome (MAS)/secondary hemophagocytic lymphohistiocytosis (HLH), which however was not abundantly present in bone marrow aspirate and the patient did not fulfill the classification criteria for MAS/HLH (triglycerides 0.72 mmol/l, fibrinogen 3.8 g/l, platelets 200 × 10^9^/l) (8). Heart ultrasonography was repeatedly normal, including at convalescence 24 days after disease onset, and the patient didn't fulfill diagnostic criteria or classical or incomplete Kawasaki disease (9). The patient was administered intravenous methylprednisolone (2 mg/kg/day, tapered over 6 days), 400 mg/kg intravenous immunoglobulins, and prophylactic nadroparin. This therapy lead to improvement of clinical symptoms with full recovery of consciousness by day 11. Her laboratory parameters normalized, except a mild hepatopathy likely related to the combined antibiotic therapy. The patient was discharged from the hospital on day 15. At follow-up 10 days after the discharge from the hospital she showed further decrease in liver enzymes, normal echocardiography, isolated non-malignant arrythmia on ECG suggestive of gradual recovery from myocarditis and displayed no clinical or laboratory signs of inflammation and had no subjective complaints. One month later the hepatopathy was resolved and the patient showed no signs of symptom recurrence.

**Figure 3:**
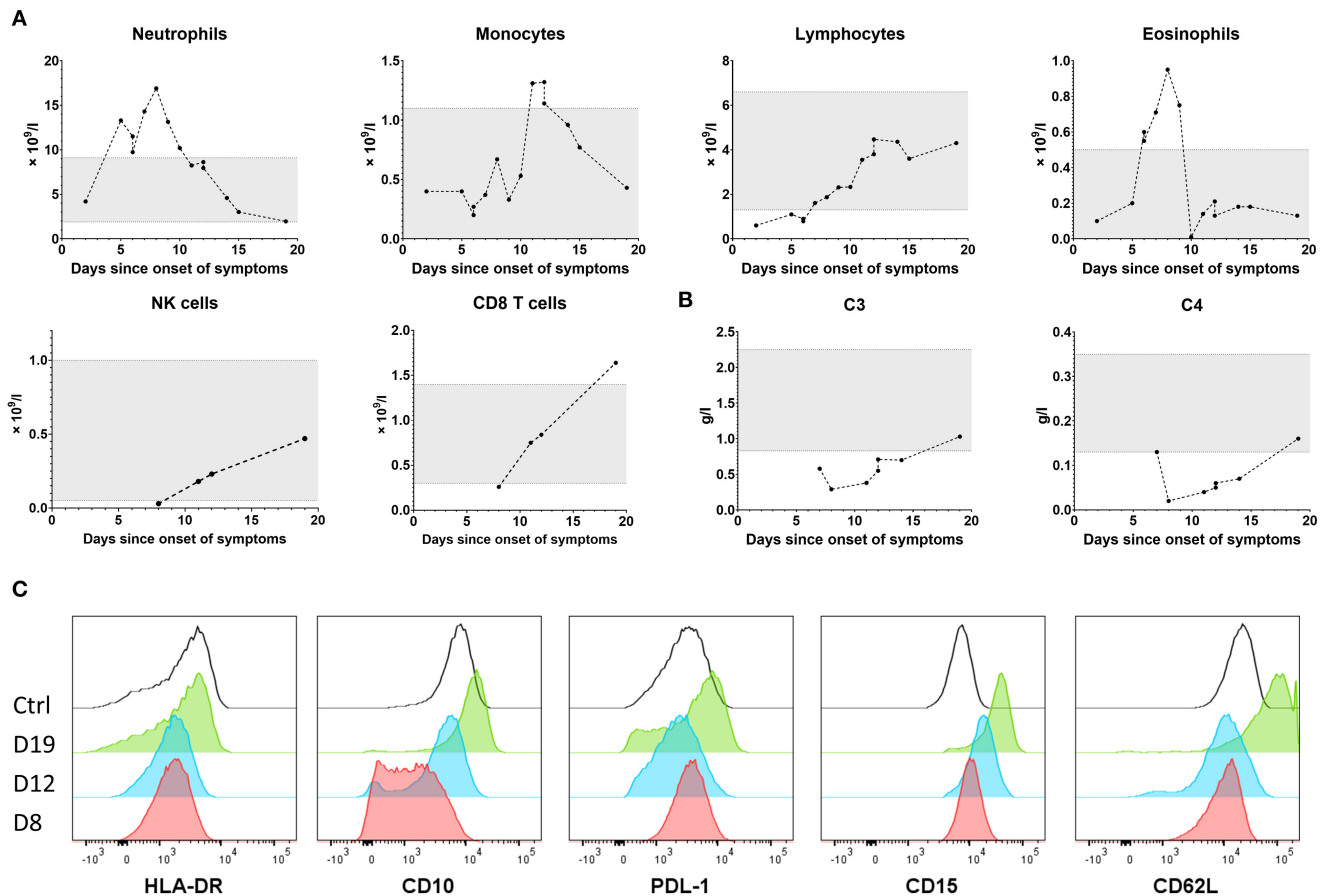
Leukocyte subpopulations over the course of the disease **(A)**. Complement protein C3 and C4 levels over the course of the disease **(B)**. Neutrophil phenotype on day 8 (fulminant disease), 12 and 19 (recovery) in comparison to a healthy control **(C)**.

Interestingly, despite massive elevation of CRP and PCT, the patient's serum IL-6 peaked at 215 pg/ml—for comparison, adult patients with severe course of COVID-19 frequently reached IL-6 levels in the thousands. Soluble IL-2 receptor, produced primarily by activated mononuclear cells, was remarkably high, although both monocytes and lymphocytes were normal on day 8 and increased only slightly between days 10 and 15 (Figure 3A). The elevation of neutrophils and lymphopenia we saw are well-established as negative prognostic markers of COVID-19 in adults, however the role of her elevated eosinophils remains elusive, as some reports suggest eosinophils in severe cases are decreased (10).

In addition to the elevation of granulocytes and low CD8 T cell and NK cell counts, we also illustrate the pivotal role of innate immunity in defense against SARS-CoV-2 by the depletion and gradual recovery of complement (C3, C4 proteins, Figure 3B). Even though we saw no direct sign of immune-complex mediated disease, abdominal pain can be one of the symptoms of immune complex-mediated IgA vasculitis.

We additionally show significant changes in neutrophil phenotype (Figure 3C) during the course of the disease, such as a decrease in neutrophil HLA-DR expression, as previously described in COVID-19 (11). For the first time in PIMS-TS we show that CD10, a marker of neutrophil maturation and immunosuppressive function (12), was markedly decreased during the worst clinical symptoms, suggesting efflux of young, active neutrophils from the bone marrow, but together with the inhibitory molecule PD-L1 and CD15, a marker of activation in mature neutrophils, reached supra-normal expression during recovery as these cells matured. The adhesion molecule CD62L was low during the most fulminant disease, but its expression recovered at convalescence. Together, these data suggest full activation and engagement of neutrophils during PIMS-TS with compensatory contraction of the response and contra-regulation of neutrophil phenotype during recovery.

## Discussion, Take-Away Lessons

In summary, our patient developed abdominal discomfort, systemic inflammatory response to SARS-CoV-2 infection in absence of other infectious pathogens. Unlike in some recently published patients (13), our patient did not fulfill diagnostic criteria for Kawasaki disease and instead displayed some but not all hallmarks of secondary MAS/HLH, fulfilling entirely the case definition criteria of PIMS-TS as proposed by both the Royal College of Pediatrics and Child Health and the Centers for Disease Control and Prevention (14, 15). Her clinical status quickly deteriorated but resolved equally promptly with corticosteroids, intravenous immunoglobulins, preventive anticoagulation, and supportive therapy.

This course of the disease is congruent with data seen in the first international PIMS-TS cohort study consisting of 58 patients from the US and Europe published this week (7), where abdominal discomfort, rash, and fever were the main presenting symptoms and good response to corticosteroids and immunoglobulins was recorded. Several patients were treated with biologics such as the IL-1R antagonist anakinra or TNF-α antagonist infliximab, targeting thus more directly the innate immune system hyperactivation which seems to drive the disease pathogenesis in PIMS-TS.

## Data Availability Statement

The raw data supporting the conclusions of this article will be made available by the authors, without undue reservation.

## Ethics Statement

The studies involving human participants were reviewed and approved by Ethical Committee of the 2nd Faculty of Medicine, Charles University in Prague and University Hospital in Motol. Written informed consent to participate in this study was provided by the participants' legal guardian/next of kin.

## Author Contributions

AK conceived the study, analyzed data, and wrote the manuscript. ZP performed neutrophil immunophenotyping. JD, PP, TV, HM, and PD reviewed the manuscript and contributed primary data. AS co-conceived the study, reviewed, and edited the manuscript. All authors contributed to the article and approved the submitted version.

## Conflict of Interest

The authors declare that the research was conducted in the absence of any commercial or financial relationships that could be construed as a potential conflict of interest.
